# The Notification of Measles

**Published:** 1921-07-02

**Authors:** 


### THE NOTIFICATION OF MEASLES.

It is a generally understood fact that measles is one of the great causes of infant mortality, and that it is at the same time a preventable disease. The quotation, "if preventable, why not prevented?" may be easily applied to this situation, but in so doing the layman and the reformer must appreciate that there are singular difficulties in the path. In the last issue of the *Lancet* appear two valuable articles by responsible public health officers who discuss some aspects of the problem.

It seems to the professional man obvious that some form of notification must be adopted if we are to know where the disease is and where it can be combated, but there are two sides even to this question when we are dealing with the greater problem of prevention. The past history of measles notification throws some useful light upon it.

Measles was not compulsorily notifiable up to the beginning of 1916, but a few sanitary authorities had, under the Act of 1889, added the disease to their local list. The serious outbreaks which occurred among troops, particularly colonials, about that time resulted in an Order made by the Local Government Board requiring compulsory notification of the disease all over England, and coincidently were made the well-remembered recommendations concerning home visiting, nursing, and provision of medical assistance, grants being promised to assist in the local expenses involved. The result of such measures as were thereby put into force had, at the termination of war, to be carefully reviewed, and ultimately the Ministry of Health, as the Board's successor, announced that continuance of such means was not the best way to deal with the measles problem, and in December 1919 measles became no longer notifiable in this country.

Explanation of this action was set down in the report of the Chief Medical Officer of the Ministry for the ensuing year. The stated reasons were that (1) notification of the first case in a house attended by a medical practitioner, and of each case in a household by the parents of the patient, is not an essential factor in a practical scheme for reducing mortality from measles; (2) that the sanitary authorities most active in this work have still to rely in large measure on other means than notification for finding out cases of the disease—*e.g.* through reports of health visitors and from schools, with the result that notification was redundant; (3) that, on the other hand, in certain districts nothing followed notification, and it was difficult to justify the expenditure of notification fees.

Thus to a large extent we are back at the beginning, but there is the difference that grants in aid of measles prevention work have been continued and the Ministry have shown their willingness to assist administratively and financially any apparently good schemes for mortality reduction which local authorities may be putting into practice.

In this way, although general compulsory notification is still in abeyance, yet it can still be brought into force locally at the Ministry's discretion; and in a number of areas, notably Paddington as described by Dr. Dudfield in the *Lancet*, this course has been followed. Elsewhere varying grades of surveillance are being exercised, and all the time valuable experience is being gained.

The chief point is, however, that not only the success or failure of the schemes, in round figures, should be given, but that the finer administrative details should be minutely exposed and discussed. It is therefore to be urged that medical officers of health will follow the lead to which the *Lancet* opens its pages and give full publicity to whatever plans have been adopted. It is in this and corresponding methods of open discussion that we shall get at the root of one of the greatest problems of preventive medicine in this country.

